# Modeling and optimization of atomic layer deposition processes on vertically aligned carbon nanotubes

**DOI:** 10.3762/bjnano.5.25

**Published:** 2014-03-05

**Authors:** Nuri Yazdani, Vipin Chawla, Eve Edwards, Vanessa Wood, Hyung Gyu Park, Ivo Utke

**Affiliations:** 1 Nanoscience for Energy Technology and Sustainability, Department of Mechanical and Process Engineering, ETH Zürich, Zürich CH-8092, Switzerland; 2 Laboratory for Nanoelectronics, Department of Information Technology and Electrical Engineering, ETH Zürich, Zürich CH-8092, Switzerland; 3 Laboratory for Mechanics of Materials and Nanostructures, EMPA, Thun CH-3602, Switzerland

**Keywords:** atomic layer deposition, vertically aligned carbon nanotubes, continuum diffusion model, conformal coating guidelines, titania, TiO2

## Abstract

Many energy conversion and storage devices exploit structured ceramics with large interfacial surface areas. Vertically aligned carbon nanotube (VACNT) arrays have emerged as possible scaffolds to support large surface area ceramic layers. However, obtaining conformal and uniform coatings of ceramics on structures with high aspect ratio morphologies is non-trivial, even with atomic layer deposition (ALD). Here we implement a diffusion model to investigate the effect of the ALD parameters on coating kinetics and use it to develop a guideline for achieving conformal and uniform thickness coatings throughout the depth of ultra-high aspect ratio structures. We validate the model predictions with experimental data from ALD coatings of VACNT arrays. However, the approach can be applied to predict film conformality as a function of depth for any porous topology, including nanopores and nanowire arrays.

## Introduction

Recent advances in the synthesis and processing of carbon nanotubes (CNTs) have enabled the prospect of their integration into existing technologies that exploit the high surface area of mesoporous ceramic films [1]. Over the last 10 years, ceramic coated CNTs have been applied in battery [2–5] and supercapacitor electrodes [6–12], fuel cells [13], and sensors [14–17]. For many of the proposed applications of these CNT/ceramic hybrids, the performances of the devices depend crucially on the thickness and conformality of the ceramic coating of the CNTs. Atomic layer deposition (ALD) is a highly attractive option for coating CNTs because it enables a wide range of ceramics and metals to be deposited conformally on arbitrary surface topologies with precise control of layer thickness [1,18].

However, vertically aligned CNT (VACNT) arrays present a complex surface and topology for ALD that requires new processing strategies. First, the graphitic surface of a pristine CNT is chemically inert, and provides no bonding sites for the nucleation of ceramics, which prevents the conformal coating of the CNT without prior functionalization [19–24]. In practice, however, chemical vapor deposition (CVD) grown CNTs are prone to a sufficient density of surface defect sites to allow for the nucleation of the ceramic at discrete points along the surface of the CNT. The ceramic then grows from these nucleation sites until it overlaps with ceramic from a neighboring site. In this way, a conformal coating forms on the surface of the nanotube [16,25–27]. While the number of cycles required to arrive at a state of complete coverage depends on the density of defect sites, 50–100 cycles are typically sufficient for a conformal coating of CVD-grown CNTs.

**Figure 1:**
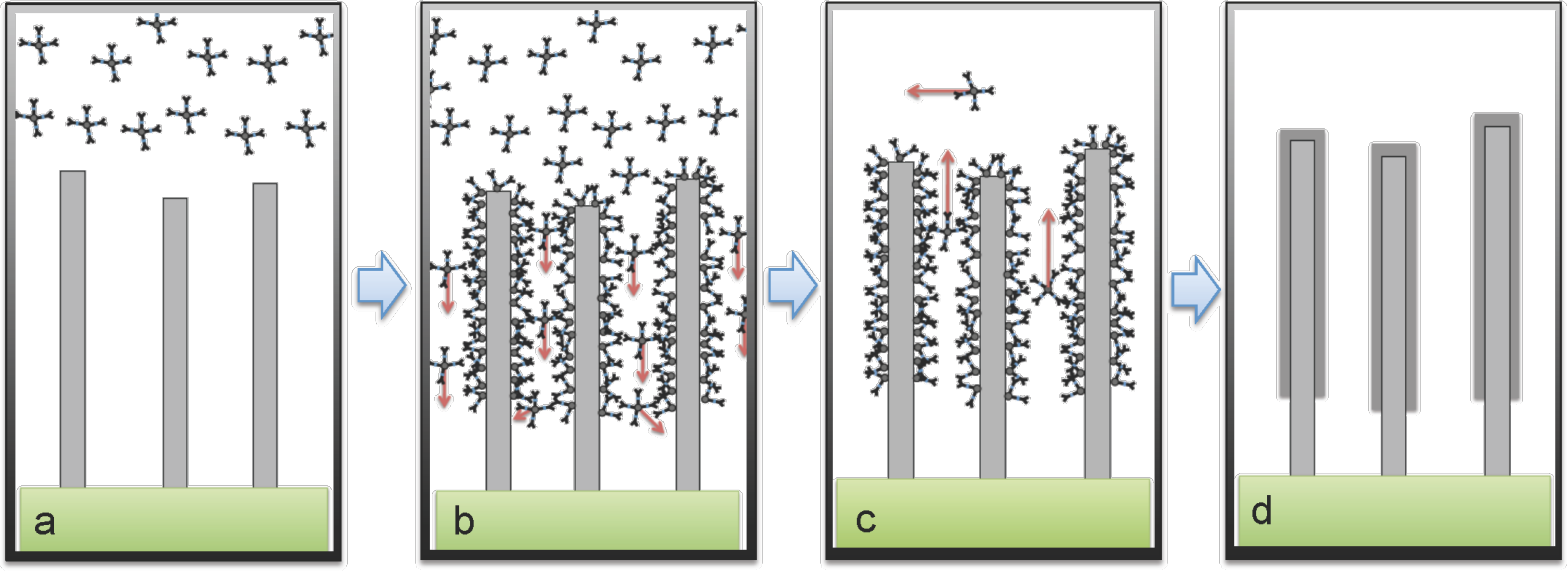
Illustration of one ALD cycle on a VACNT array. Upon exposure to the precursor gas (a: bulk gas diffusion), precursor molecules diffuse into the CNT array and adsorb onto available sites (b: confined diffusion and adsorption). After the defined precursor exposure time, purging of the ALD chamber removes unreacted precursor molecules from the chamber (c). After the oxidization pulse, a thin layer of oxide is formed on the surface (d).

Second, the vertically aligned nature of the CNT arrays presents a challenge for conformal coatings of uniform thickness. The penetration of the deposited oxide into the VACNTs is often limited as illustrated in Figure 1. Under the pressure and temperature conditions of typical ALD processes, gas phase collisions among the precursor molecules in the pores of VACNT structure are far less frequent than collisions between a precursor molecule and a CNT surface. This corresponds to the free molecular regime of gas transport (i.e., Knudsen diffusion) [28–29]. Furthermore, even VACNTs of moderate lengths have very large surface areas that require a large number of precursor molecules for a monolayer coating, such that either large precursor concentrations or very long exposure times are needed to conformally coat all the way down to the bottom of the array [30]. An optimization of the ALD process can ensure the desired depositions while minimizing the use of precursor material and the deposition time [16,27]. Here we develop and validate a model to perform such optimization.

Previous modeling [31–36] of the penetration of metal oxides into nanometer-sized pores has demonstrated that the factor limiting the penetration depth of the oxide is the depth, to which the precursor molecules can diffuse in the pores and adsorb on the pore surface during the precursor exposure/adsorption step of the ALD process. The penetration depth of the oxide into the pores, *x*_p_, was shown to be proportional to the pore radius, 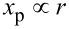 the square root of the precursor exposure time, 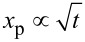 and the square root of the precursor concentration in the chamber, 
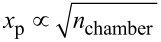
 [31–33]. For porous media, the radius of the pores will decrease from cycle to cycle, which further hinders a diffusion of the precursor molecules. In addition, in the case of VACNTs, not only will diffusion be increasingly hindered because of the cycle-to-cycle increase of the CNT radii, but the total surface area will also increase, which means that more and more precursor molecules are required to completely cover the CNTs with adsorbed precursors, as illustrated in Figure 1. Understanding how the penetration depth of the precursor varies with the ALD process parameters and radii of the CNTs could thus enable deposition recipes to be optimized to obtain a ceramic with uniform thickness to a desired penetration depth.

To this end, we develop a model that treats both the diffusion of precursor molecules and their adsorption on VACNTs during the precursor pulse. Assuming rapid adsorption of the precursor molecules, this full diffusive model can be approximated by a close form expression, equivalent to the well-established Gordon model [32]. We use the full diffusion model to investigate the effects of the various ALD process settings and physical parameters of the CNTs on the oxide growth kinetics during a single ALD cycle. Multi-cycle ALD processes are simulated, and reveal that non-uniform coating thickness as a function of depth results from the decrease in the penetration depth from cycle to cycle. The model predicts that if the precursor exposure times are scaled as a function of cycle number, uniform depositions can be achieved. Finally, we experimentally confirm our model predictions.

## Results and Discussion

### Modeling of precursor exposure/adsorption

When performing ALD on structures with a high aspect ratio, one process that limits the uniform coating could be the relatively slow diffusion of the precursor molecules within the structures. At the same time, the large surface area of the high-aspect-ratio structure requires a large number of precursor molecules such that the location and rate of adsorption is also critical to the coating kinetics. Therefore to understand how ALD process parameters influence the coatings of high aspect ratio structures, we develop a model based on a continuum diffusion approach that also takes into account adsorption of precursor molecules.

In this model, we exclude the discrete nucleation phase of the ALD process on the CNTs. That is, we assume that a layer of ceramic is already deposited on the nanotubes, which may typically require 50–100 ALD cycles to achieve. The model may thus underestimate the penetration depth of the ceramic for a low number of ALD cycles, but it should be qualitatively accurate for high cycle numbers.

### Diffusion model of the precursor adsorption kinetics

In ALD, the precursor is pulsed into the chamber diluted in a carrier gas, such as N_2_ or Ar. An overall chamber pressure of the order of several millibar are typical for ALD processes*,* with precursor number densities ranging from 10^13^–10^15^ cm^−3^*.* As the mass of the precursor molecules, *m*_p_, and their molecular diameter are in general much larger than those of the carrier gases, the diffusion of the precursor molecules within the VACNT array is far slower than that of the carrier gas. Here, we assume a mechanical equilibrium between the porous region of the CNT arrays and the rest of the ALD chamber such that total pressures within and outside the CNT arrays are quickly equilibrated as a result of fast distribution of the carrier gas. We can thus model the transport kinetics as a diffusive process of the precursor species in a porous medium initially filled with the carrier species.

Assuming a negligible variation of the precursor partial pressure in the transverse directions of the CNT arrays, we can represent the precursor number density per unit volume, *n*(*x*,*t*), as a function of time, *t*, and the distance into the CNT array, *x*. The function *n*(*x*,*t*) obeys the following transport equation:

[1]
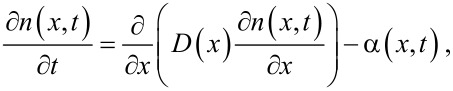


where *D*(*x*) is the diffusion coefficient of the precursor molecules inside the CNT array, and α(*x*,*t*) is a loss term that corresponds to the adsorption of precursor molecules onto available bonding sites and their subsequent chemical bond formation (chemisorption).

The adsorption rate per unit volume, α, is modeled to be proportional to the number of precursor molecules striking the CNT surface per unit area, i.e., the impingement rate, *I*(*x*,*t*), to the fraction of the CNT surface area available for the precursor adsorption, *f*(*x*,*t*), and to the probability, at which an impinging precursor molecule adsorbs and reacts on an adsorption site, i.e., the reactive sticking coefficient, Γ:

[2]
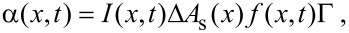


where Δ*A*_s_ (cm^−1^) is the surface area per unit volume of the CNT array. We take the impingement rate from gas kinetics theory, in which for an ideal gas,

[3]
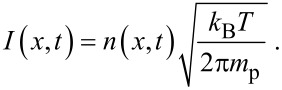


The fraction of the surface area that is available for adsorption is given by

[4]
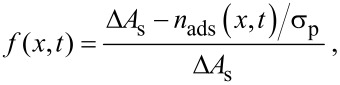


where σ_p_ is the maximum number of adsorbed precursor molecules per unit area, and

[5]
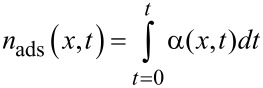


is the total number of precursor molecules per unit volume already adsorbed at time *t*.

Substituting Equation 2 into Equation 1, we simulate Equation 1 by using a finite difference method. Both time and space are discretized into points separated by Δ*t* and Δ*x* respectively,

[6]
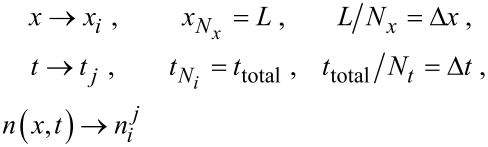


**Figure 2:**
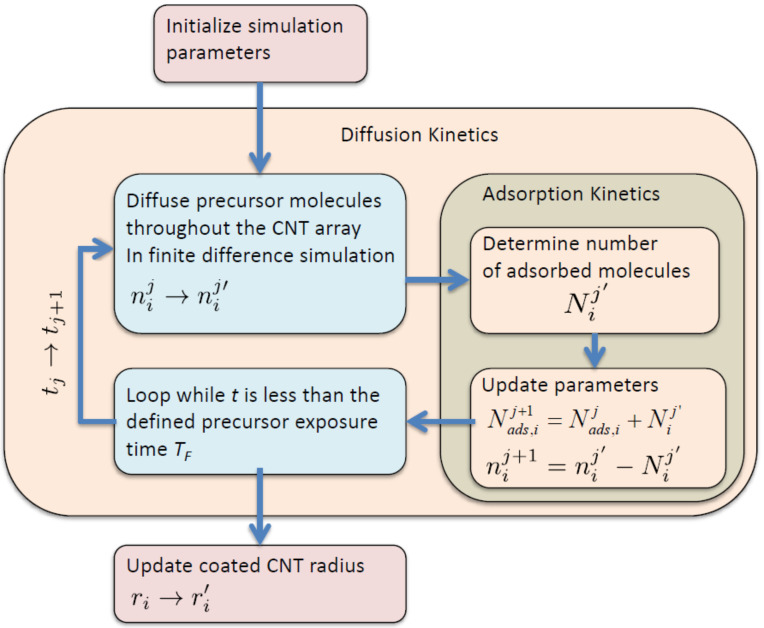
Flow chart of the precursor exposure/adsorption simulation for one ALD cycle.

Since the adsorption kinetics typically proceed at a much faster rate than the diffusion of precursor molecules, we also decouple Equation 1 in the simulation into two parts. To update the simulation parameters at time *t_j_*_+1_, the diffusion process is advanced first in the simulation, generating a concentration profile given by 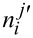. The adsorption kinetics are then solved explicitly over the period t*_j_* → t*_j_*_+1_ and 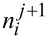 and 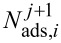 are then computed accordingly, as described in more detail below. A flow chart of the simulation of one ALD cycle is shown in Figure 2.

For the precursor pulse portion of the ALD cycle, we use the initial condition that the CNT array is devoid of any precursor molecules, 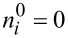. The system has a closed boundary at *x = L* (the bottom of the VACNT array), which is implemented by a first order Neumann boundary condition, *dn*/*dx* = 0. At *x =* 0 (the top of the VACNT array) we apply 
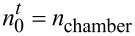
, where *n*_chamber_ is the concentration of the precursor molecules in the ALD chamber. For the purge portion of the ALD cycle, we take as the initial condition the final state of the system at the end of the precursor pulse portion. The boundary condition at the top of the CNT array (*x =* 0) is also changed to reflect that there are no precursor molecules in the chamber (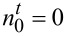).

### Precursor adsorption kinetics

At each time step *t_j_*, the amount of precursor molecules that adsorb onto vacant adsorption sites during the time *t_j_* → *t_j_*_+1_ is calculated for each discrete void volume element Δ*V_i_*, where Δ*V_i_* ≡ Δ*V*·Δ*x*, at position *x_i_*. We let *N_i_*(*t*) represent the number of precursor molecules per projected area in Δ*V_i_*, continuously in time from *t_j_* → *t_j_*_+1_, where 
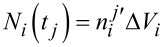
. According to Equation 2, *N_i_*(*t*) should satisfy:

[7]
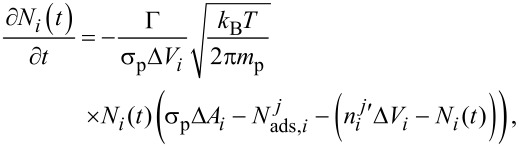


where Δ*A_i_* ≡ Δ*A*_s_·Δ*x*. Solving Equation 7 and applying the initial condition 
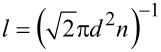
 gives

[8]
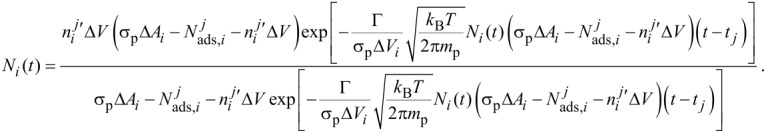


Equation 8 is then used to calculate the total amount of precursor molecules that are adsorbed in the volume Δ*V_i_*, giving

[9]
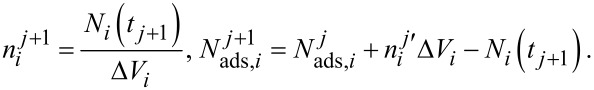


### Diffusion coefficient

From the kinetic theory of an ideal gas, the mean free path is given by 
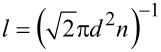
, where *d* is the molecular diameter, and *n* is the gas number density. Most precursor molecules have diameters in the range of 10^−8^–10^−7^ cm, while precursor concentrations can range anywhere from 10^13^ cm^−3^ to 10^15^ cm^−3^ with chamber pressures typically of the order of a few millibar. Even assuming partial pressures of the precursor molecule of up to 10 mbar, the mean free path calculated from kinetic theory (1.5 × 10^−4^ cm) is more than an order of magnitude greater than the average distance between the CNTs of the vertical array, which is typically smaller than 10^−5^ cm. The gas transport of this system is thus in the regime of free molecular flow, in which the effective diffusion coefficient for this porous medium is described by Knudsen diffusivity,

[10]
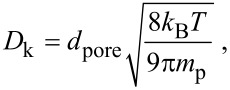


where *d*_pore_ is the average pore size of the porous medium. As a first order approximation, we use Equation 10 and replace *d*_pore_ with the average spacing between the CNTs in the array. If the CNTs have an areal density of *σ*_CNT_ (cm^−2^) and the radius *r*, the effective diffusion coefficient will be given by

[11]
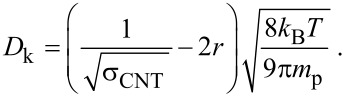


### Simulation results

Table 1 summarizes our estimates for the physical parameters of the CNTs and precursor molecules as well as the ALD run parameters that were used in the simulations. Values for the precursor parameters are estimated from titanium isopropoxide. The precursor surface adsorption density, σ_p_, is estimated to be 1/*V*_AB2_^2/3^, where *V*_AB2_ is the volume of a deposited oxide unit, here determined by the dimensions of the titanium dioxide unit cell.

**Table 1:** Default parameters for the simulation.

simulation parameter	value

height of the VACNTs	10^−2^ cm
initial average CNT diameter	10^−6^ cm
areal density of the CNTs (σ_CNT_)	10^10^ cm^−2^

diameter of precursor molecule	10^−7^ cm
mass of precursor molecule (*m*_p_)	4.7 × 10^−22^ g
precursor surface adsorption density (σ_p_)	10^14^ cm^−2^
volume per oxide unit (AB_2_)	1.4 × 10^−22^ cm^3^
concentration of precursor vapor (*n*_chamber_)	5 × 10^15^ cm^−3^
reactive sticking coefficient	0.01

ALD deposition temperature	500 K
precursor exposure time	50 ms
purge time	50 ms

**Figure 3:**
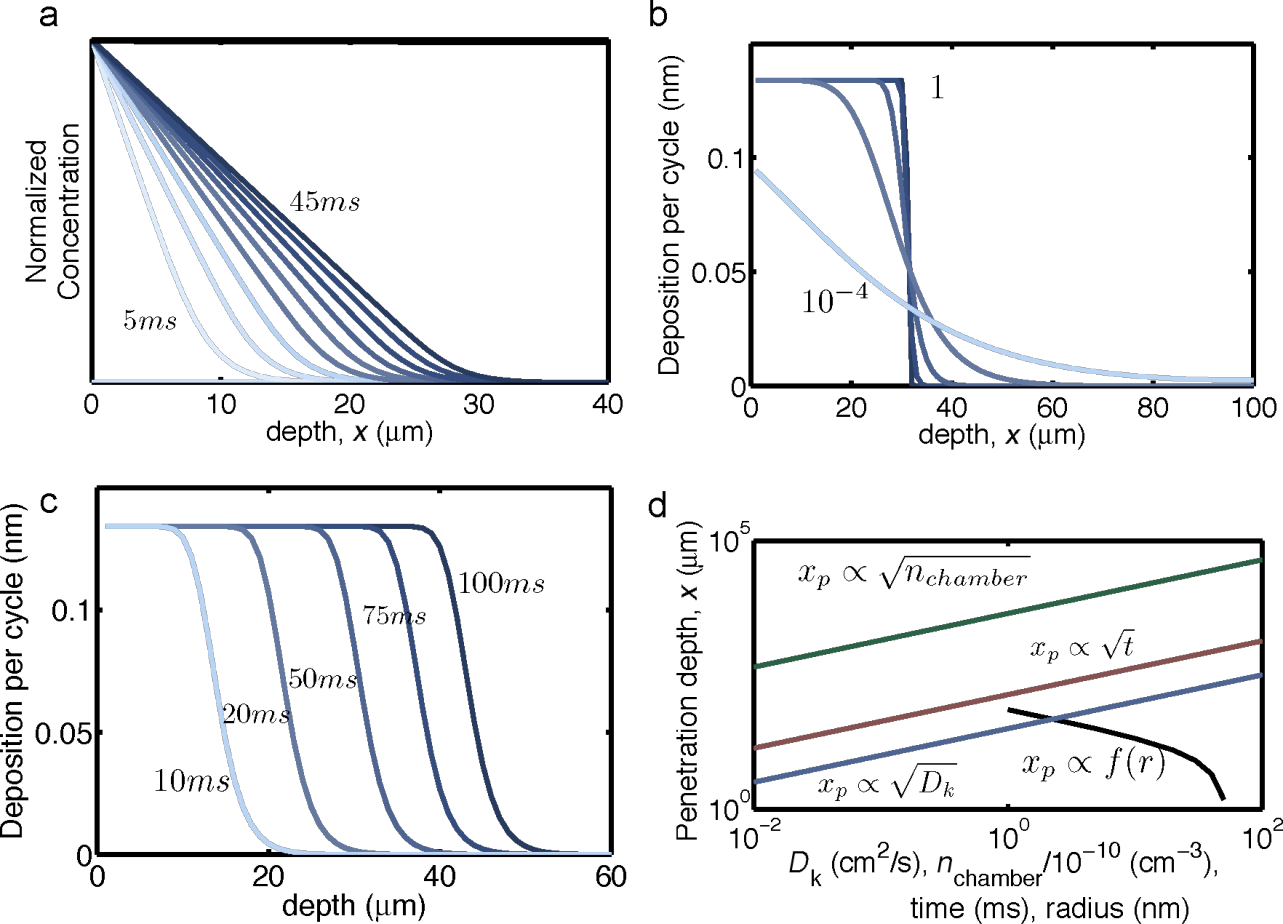
Results of the precursor adsorption kinetics simulation while using the parameters defined in Table 1. The concentration of precursor molecules as a function of depth at several time steps (0 to 50 ms, with 5 ms intervals) within the CNT array is plotted in (a). The effect of the reactive sticking coefficient is demonstrated in (b), where the deposited oxide thickness per cycle is plotted versus the depth in the array, after 50 ms exposure. In (c) the effect of the precursor exposure time is plotted. In (d), the penetration depth of the oxide is plotted as a function of the precursor concentration, exposure time, Knudsen diffusion coefficient, and radius of the CNTs with a reactive sticking coefficient of 10^−2^. The penetration depth clearly depends on the square root of the precursor concentration, exposure time, and diffusion coefficient, but has a nontrivial dependence on the radius of the CNTs.

By using the parameters defined in Table 1, the concentration of the precursor as a function of the depth within the VACNT array is plotted at various times during the exposure to the precursor of 50 ms in Figure 3a. Immediately after its introduction into the ALD chamber, the precursor can easily penetrate the CNT arrays down to 10 μm. The penetration becomes limited, however, to 30–40 μm even after the full 50 ms of exposure, which is attributed to decreasing flux of the penetrating precursor as a function of depth into the array, ∂*n*/∂*x*.

In Figure 3b, the thickness of oxide coated per cycle is plotted as a function of depth for various reactive sticking coefficients ranging from 10^−4^ to 1. For relatively large coefficients (from about 10^−2^ to 1), the penetration depth is not greatly affected by the reactive sticking coefficient, which indicates that the impingement rate of the precursor molecules on the CNT surface is large compared to the rate, at which they diffuse into the CNT array. In Figure 3c the thickness of oxide coated per cycle is plotted with respect to depth for various precursor exposure times. The thickness of the coating deep inside the CNT array increases with the exposure time, as the precursor molecules have more time to diffuse down to available adsorption sites.

The maximum growth per cycle of about 0.13 nm is rather large compared to typical ALD deposition rates for titanium dioxide. In principle, one can modify the precursor surface adsorption density to achieve more reasonable deposition rates. The two extreme limits for the maximum growth per cycle would be the limits, at which the density is either determined by the oxide volume, σ_p_ = 1/*V*_AB2_^2/3^ as used for the data above, or by the diameter of the precursor molecules, σ_p_ = 1/π*r*_p_^2^ where *r*_p_ is the radius of the precursor molecule.

The penetration depth of the oxide, *x*_p_ (defined as the depth, at which the thickness of the coating is equal to half of its maximum value), is characterized while varying the exposure time, precursor concentration, diffusion coefficient, and the radius of the CNTs (Figure 3d). The penetration depth is found to be proportional to the square root of the exposure time, the precursor concentration, and the diffusion coefficient, which is consistent with previous studies [32–33]. Interestingly, the penetration depth shows a non-trivial dependence on the radius of the CNTs. The radius of the CNTs has an impact on two parameters in the diffusion/absorption process. As the radius of the CNTs is increased, the gap distance between the CNTs decreases, which reduces the diffusion coefficient given in Equation 11. In addition, as the radius of the CNTs increase, the overall surface area per unit volume, Δ*A*_s_, also increases, such that more precursor molecules are adsorbed per unit length. The combination of slower diffusion and increased demand of precursor supply to fully coat the surface has a strong impact on the extent, to which the oxide can penetrate into the CNT array.

### Approximation to the model assuming rapid adsorption

Under the condition that the adsorption rate of precursor molecules is much faster than the diffusive transport through the CNT array and with a reactive sticking coefficient close to one, it can be assumed that all the precursor molecules adsorb at the first encounter with an unoccupied adsorption site [32]. In this scenario, the adsorption sites are filled up linearly in the *x* direction, and the precursor concentration is zero at *x*_p_, since all of the precursor molecules arriving at *x*_p_ are immediately adsorbed. Furthermore, the precursor diffusion flux throughout the array up to *x*_p_ must be constant, since precursor molecules are adsorbing at a fixed rate at *x*_p_. Thus, given that the concentration at *x =* 0 is fixed at *n*_chamber_, Fick’s law gives the precursor diffusion flux as

[12]
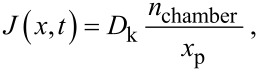


or in terms of the number of precursor molecules per unit projected area, *N*,

[13]
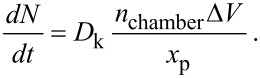


As precursor molecules continuously flow into the CNT array, the change in *x_p_* can be expressed as

[14]
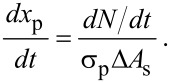


Substituting Equation 13 into Equation 14 and a subsequent integration gives

[15]
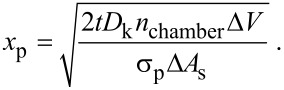


This result is equivalent to the expression given by Gordon et al. [32] for tubular pores taking Equation 10 for *D*_k_ and considering that

[16]
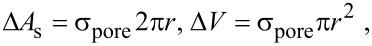


under the condition that the pore radius, *r*, is much smaller than the depth of the pores. Equation 15 thus gives an analytical approximation for the oxide penetration depth valid for any porous topology with high aspect ratio provided that the reactive sticking is close to one (Γ ≈ 1). It only requires the determination of (1) an effective diffusion coefficient related to the porosity, constrictivity, and tortuosity of the porous medium and of (2) the surface area and void volume per unit volume.

For an array of CNTs the surface area and void volume per unit volume are given by

[17]
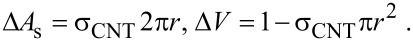


Substituting Equation 16 and Equation 11 into Equation 15 then gives the final expression for the penetration depth of one ALD cycle,

[18]
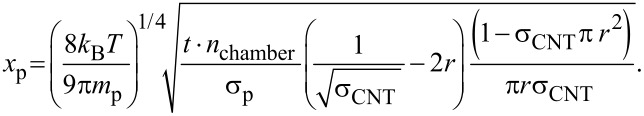


**Figure 4:**
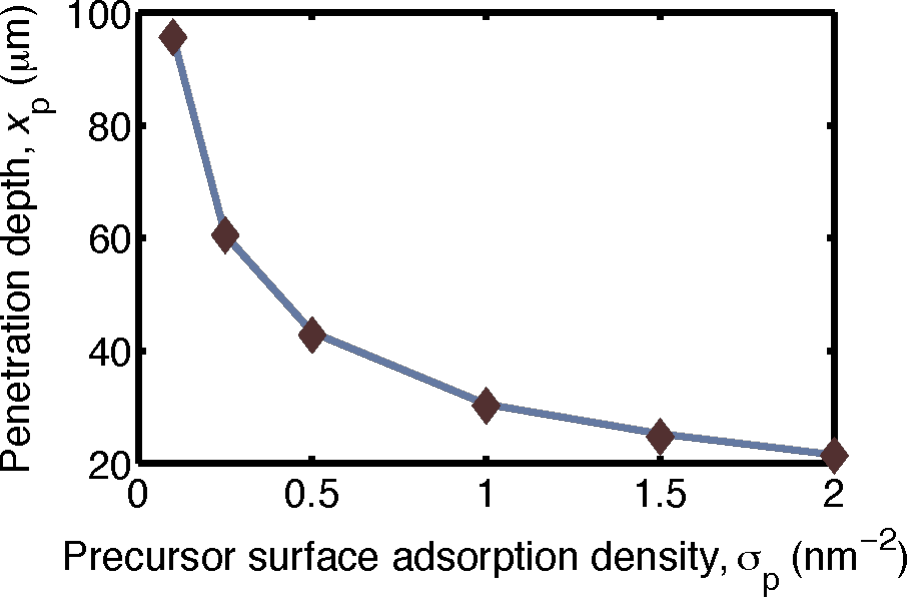
Comparison of the simplified model (Equation 18) and the simulation of the full diffusion model (solid line) while using a reactive sticking coefficient Γ = 1, as function of the varying precursor surface adsorption density. The penetration depths given by both methods are in agreement.

This simplified expression is in agreement with the simulations of the full diffusion model above. In Figure 4, the penetration depth of the oxide is plotted by using both the simulation and the simplified expression while using the parameters in Table 1 with varying precursor adsorption site densities. The results when using Equation 18 are in agreement with the simulation output.

### Multiple cycle growth kinetics

**Figure 5:**
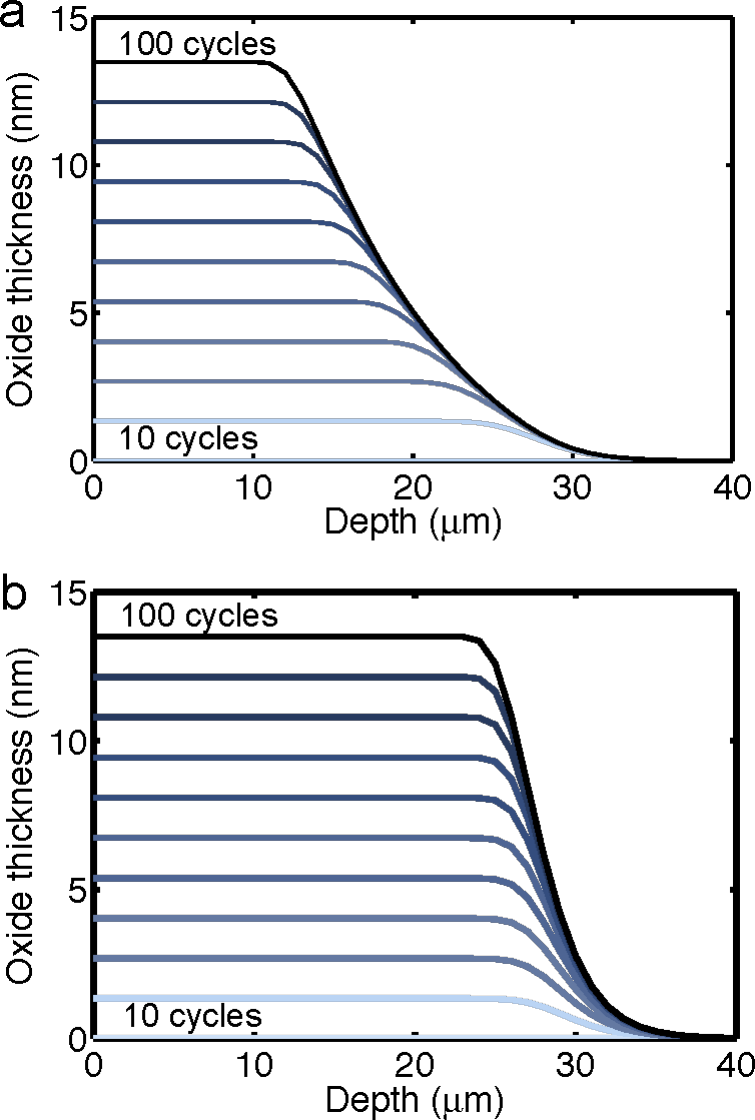
Plot of deposited oxide thickness with respect to the VACNT depth for a multi-cycle ALD process, determined by the model with the parameters in Table 1. (a) Fixed precursor exposure time (50 ms). (b) Scaled exposure time according to Equation 19.

The dependence of the penetration depth for the precursor adsorption on the radius of the CNTs (Equation 18) implies that the depth of the ALD deposition will decrease from cycle to cycle. Depositions on VACNT arrays that utilize an ALD process with fixed deposition parameters typically display a decreasing coating thickness as a function of the depth within the array. From the results of our modeling, it is clear that this depth profile of the coating predominantly occurs not through an uneven deposition profile from each individual cycle but rather from a cycle-to-cycle variation in the penetration depth of the oxide coating. In Figure 5a, the oxide thickness with respect to depth is plotted according to results of the multi-cycle ALD simulation. These findings suggest that, in order to achieve truly uniform coatings on a high-aspect-ratio structure, one must scale the ALD parameters from cycle to cycle. Although changing the precursor concentration and temperature is generally impractical, the precursor exposure time can be changed rather easily. If cycle zero has an exposure time, *t*_0_, with CNTs of radius, *r*_0_, the subsequent exposure times required to always reach the same penetration depth are given as:

[19]
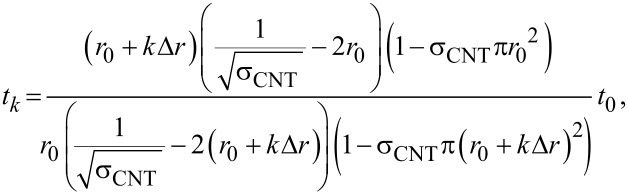


where Δ*r* is the thickness of oxide deposited per cycle, and *k* is the cycle number. This scaling is simulated, and shows (Figure 5b) a more uniform coating thickness as a function of depth compared to the coating obtained with a constant precursor exposure time (Figure 5a). This result suggests a clear ALD process protocol to achieve a uniform coating of high aspect ratio structures.

## Experimental

To qualitatively compare the results of the modeling to actual depositions, VACNT samples are prepared and coated with titanium dioxide and aluminum oxide. To synthesize our VACNTs, a 3-nm-thick catalyst layer of iron on top of a 20-nm-thick layer of aluminum is deposited through electron beam evaporation onto a silicon wafer. The VACNTs are then grown by chemical vapor deposition in a cold-wall CVD system. The catalyst-covered substrate is annealed for 10 min at 725 °C in a flowing environment of Ar (400 sccm) and H_2_ (600 sccm) in order to reduce the iron oxide to metallic iron. After this reduction annealing, C_2_H_4_ (250 sccm) as a carbon precursor is supplied for 5 min, which results in the growth of vertically aligned multiwalled CNTs, 50–90 µm in height. By using scanning electron microscopy (SEM), the distribution of CNT diameters is measured, and the bare CNT radii are found to be 6.3 ± 0.2 nm. Titanium dioxide is deposited on the CNTs by using a custom-built ALD system. The depositions are performed at 225 °C with a precursor mixture of titanium isopropoxide (Ti{OCH(CH_3_)_2_}_4_, TTIP) heated to 90 °C and water vapor at 40 °C. One ALD cycle consists of a 5 s long pulse and 40 s long hold of TTIP, followed by a 0.5 s long pulse and 40 s long hold of water vapor, with 60 s long Ar purges in between the two pulses. Aluminum oxide is deposited on the CNTs by using a Picosun ALD system. The depositions are performed at 200 °C. Trimethylaluminum (Al_2_(CH_3_)_6_, TMA), held at room temperature, is used as the precursor. One ALD cycle comprises of a 0.2 s long pulse of TMA, which is followed by a 5 s long N_2_ purge and two sequential 0.2 s long water vapor pulses. For the scaled deposition, the TMA pulse duration is modified while the remainder of the ALD procedure remains unchanged.

**Figure 6:**
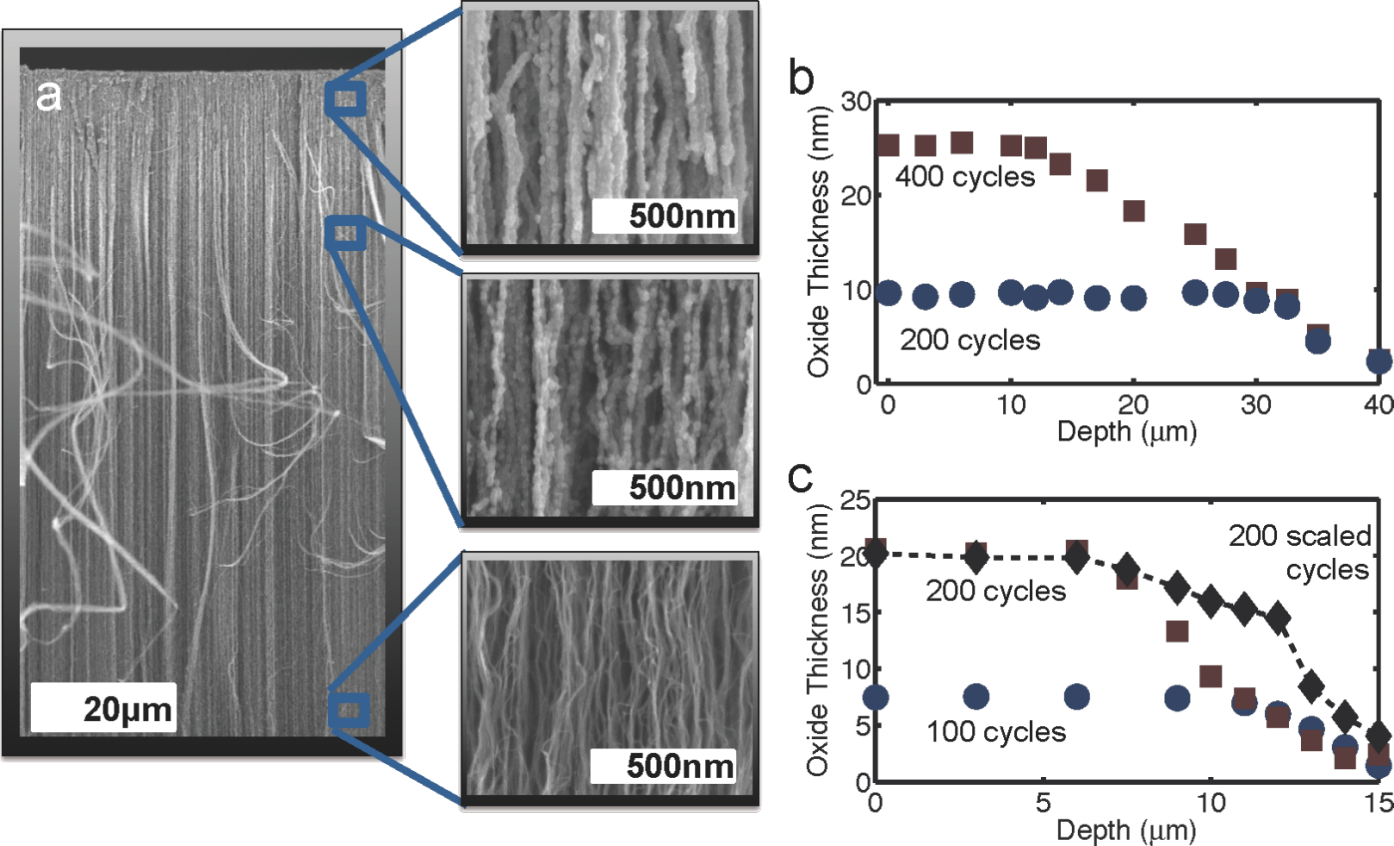
Experimental results for TiO_2_ coated VACNTs. (a) An SEM image of a VACNT array coated with 400 cycles of TiO_2_ is shown with close-up SEM images at depths of ca. 5, 25, and 80 μm. At 5 μm, a thick and conformal coating of TiO_2_ is evident. At 25 μm the oxide coating is visibly thinner, and exposed CNTs are visible, at which the oxide beads have not merged, yet. Down at approx. 80 μm, only small nucleations of the oxide are noticeable. The TiO_2_ thickness on the CNTs (determined by the measurement of the coated-CNT diameter distribution) as a function of depth is plotted in (b) for samples coated with 200 (circles) and 400 (squares) cycles. Error bars are indicated by the size of the points. Plot (c) shows the thickness of aluminum dioxide as a function of depth for the samples prepared with 100 cycles (blue circles) and 200 cycles (red squares), for which the precursor exposure time was fixed at 0.2 s, along with a plot for a sample prepared where the precursor exposure time was scaled as a function of ALD cycles between the 100th and 200th cycles (black diamonds).

Figure 6 shows SEM images of a sample processed with 400 ALD cycles. Near the top of the VACNT array, the CNTs are clearly coated conformally with a thick layer of TiO_2_. Further down the array, the TiO_2_ coating becomes thinner, until only a few nucleation points of the oxide are visible on the CNTs. The rough surface of the oxide indicates the initial stages of nucleation. On the bare CNTs, the precursor adsorbs only onto discrete defect sites on the CNTs, and these nucleation centers expand with subsequent cycles, until they merge with one another.

The thickness of the deposited oxide as a function of the depth within the array is determined by measuring the diameter distributions of the coated nanotubes at various depths. This is carried out for two samples, one processed with 200 ALD cycles and the other processed with 400 cycles. The 200-cycle sample has a roughly constant oxide thickness of about 10 nm from the top of the array down to 28 μm, whereas the 400-cycle sample has an average oxide thickness of about 25 nm from the top of the array down to ca. 12 μm. At depths greater than approx. 30 μm, both samples have roughly the same oxide coating thickness. This confirms the model prediction that non-uniform oxide thickness does not arise as a result of a non-uniform deposition profile per cycle but rather from the decrease of the penetration depth of the oxide coating from cycle to cycle. A comparison of experimental and simulation results shows that the model scales correctly with the ALD cycle number. From the experimental data in Figure 6b, we find that for 400 cycles we have *x*_p_ ≈ 12 μm and *r*_400_ ≈ 28 nm, while for the 200 cycle sample *x*_p_ ≈ 28 μm and *r*_200_ ≈ 13 nm. By using Equation 18 we can calculate *x*_p_(*r*_200_)/*x*_p_(*r*_400_) ≈ 2.1, which is very close to the measured value of (28 μm)/(12 μm) ≈ 2.3. One can utilize similar measurements to develop ALD recipes that can provide a uniform coating of the oxide up to a desired penetration depth. The parameter, Δ*r*_,_ required in Equation 19 can be extracted from the measurements of the oxide thicknesses for the coatings that result from two different numbers of cycles. The overall penetration depth can then be tuned, since it scales with the square root of the precursor exposure/adsorption time (see Equation 18).

To experimentally validate the pulse time scaling procedure developed from the model, we perform depositions of aluminum oxide on VACNT samples, during which we attempt to keep the penetration depth of each ALD cycle constant over one hundred cycles. In Figure 6c, the oxide thickness as a function of the depth within the array is plotted for samples coated with 100 and 200 cycles of aluminum oxide by using a fixed exposure time, and for one sample with 200 cycles, for which the exposure time from the 100th to the 200th cycle was scaled according to Equation 19. As expected, a uniform thickness up to *x*_p_ ≈ 12 μm is obtained for the 100-cycle sample, while a uniform thickness down to *x*_p_ ≈ 7 μm is obtained for the 200-cycle sample. From the plots, we determine Δ*r* ≈ 0.12 nm and we approximate σ_CNT_ ≈ 10^9^ cm^−2^. For the number of cycles investigated here, Equation 19 can be approximated by *t*_k_ = *t*_0_ + *t*_0_*k*Δ*r*/*r*_0_. By scaling of the pulse times between the 100th and 200th ALD cycles, the coating thickness remains roughly uniform down to *x*_p_ ≈ 12 μm, as desired. The decrease in thickness at depths smaller than about 12 μm, as well as the thicker than expected coatings at depths greater than ca. 12 μm, are attributed to the fact that in the ALD system used, the pulse times are rounded to the nearest tenth of a second, which causes some ALD cycles during the scaling steps to be longer or shorter than desired. This result indicates that the scaling the pulse times is a viable method to obtain uniform coatings down to specific depths on high aspect ratio structures.

## Conclusion

Our model and experiments indicate that limited penetration depth and non-uniformity of the ALD coatings of VACNTs result from the combination of slower diffusion and increased demand for precursor supply, effects that become increasingly important with each ALD cycle. This finding allows us to propose and subsequently validate that uniform ALD coatings to a desired depth within a high-aspect-ratio structure can be achieved by cycle-to-cycle variation of the precursor exposure time.

For the sake of convenience, a summary of important variables that were used in the modeling is given in Table 2.

**Table 2:** Summary of important variables used in the modeling.

variable	description	unit

*n*	density of precursor molecules	cm^−3^
*n*_ads_	density of adsorbed precursor molecules	cm^−3^
*N*	precursor molecules per unit sample area	cm^−2^
*x*	distance into the CNT array	cm
*Α*	absorption rate per unit volume	cm^−3^s^−1^
*I*	impingement rate of precursor molecules	cm^−2^s^−1^
*D*_k_	diffusion coefficient of precursor molecules within the CNT array	cm^2^s^−1^
*f*	fraction of the CNT surface area available for the precursor adsorption	—
Γ	reactive sticking coefficient	—
Δ*A*_s_	surface area per unit volume	cm^−1^
Δ*V*	void volume per unit volume	—
σ_CNT_	CNT density	cm^−2^
σ_p_	adsorption site density	cm^−2^

Δ*x*	spacing of discretized points in simulation	cm
Δ*A_i_*	surface area contained in *Δx* per unit area	—
Δ*V_i_*	void volume contained in *Δx* per unit area	cm
*n_i _^j^*	density of precursor molecules	cm^−3^
*N^j^*_ads_*_,i_*	number of adsorbed precursor molecules	cm^−2^
